# The association of leisure-time physical activity and active commuting with measures of socioeconomic position in a multiethnic population living in the Netherlands: results from the cross-sectional SUNSET study

**DOI:** 10.1186/1471-2458-12-815

**Published:** 2012-09-21

**Authors:** Jeroen SL de Munter, Charles Agyemang, Lizzy M Brewster, Karien Stronks, Irene GM van Valkengoed

**Affiliations:** 1 Department of Public Health, Academic Medical Center, University of Amsterdam, Amsterdam, The Netherlands; 2 Department of Internal Medicine, Academic Medical Center, University of Amsterdam, Amsterdam, The Netherlands

**Keywords:** Ethnicity, African, Indian, Social class, Physical activity, Commuting, Transportation, Ethnic groups, Health behavior, Minority health

## Abstract

**Background:**

In most European origin populations measures of socioeconomic position are positively associated with leisure time physical activity (LTPA), this is unclear for active commuting. In addition, these associations have scarcely been studied in ethnic minority groups, who often have a high cardiovascular disease risk. Because of the expected public health potential, we assessed the relationship of active commuting and LTPA with measures of socioeconomic position across two large ethnic minority groups in the Netherlands as compared to the European-Dutch population.

**Methods:**

We included South Asian-Surinamese (*n* = 370), African-Surinamese (*n* = 689), and European-Dutch (*n* = 567) from the cross-sectional population-based SUNSET study (2001–2003). Active commuting and LTPA were assessed by the SQUASH physical activity questionnaire and calculated in square-root-transformed metabolic equivalents of task-hours/week (SQRTMET). Socioeconomic position was indicated by level of education (low/high) and occupational class (low/high). We used age-adjusted linear regression models to assess the association between physical activity and socioeconomic position.

**Results:**

Compared to the European-Dutch men, South Asian-Surinamese men engaged in lower levels of commuting activity and LTPA, and South Asian-Surinamese women engaged in lower levels of LTPA than their European-Dutch counterparts. Differences between the African Surinamese and the European-Dutch were small. We observed a positive gradient in active commuting activity for education in European-Dutch men (beta high education was 0.93, 95%CI: 0.45-1.40 SQRTMET higher versus low education), in South Asian-Surinamese men (beta: 0.56, 0.19-0.92), but not in African-Surinamese men (−0.06, -0.45-0.33, *p* for ethnicity-interaction = 0.002). In women we observed a positive gradient in active commuting activity and occupational class in European-Dutch women, and less strongly in South Asian-Surinamese and African-Surinamese women (*p* for ethnicity-interaction = 0.02). For LTPA and socioeconomic position, we observed no statistically significant interaction by ethnicity.

**Conclusions:**

The positive gradient for socioeconomic position observed in European-Dutch was less strong, in particular for active commuting, among the South Asian-Surinamese and the African-Surinamese. This indicates that the typical focus on physical activity interventions in lower socioeconomic groups could work for European-Dutch populations, but this strategy may not be entirely applicable in the ethnic minority groups.

## Background

The importance of physical activity as a means to lower cardiovascular disease risk is well established [1-4]. Arguably, lifestyle activity or habitual activity associated with daily living has the most public health potential and is of great interest to physical activity researchers at present [5]. Traditionally, physical activity interventions have focused on promoting more leisure-time activities (e.g., structured exercise programmes). But, lack of evidence for effectiveness and sustainability of any changes [6], has led to growth in interest in designing environments that are conducive to active living, for instance promoting habitual activities in leisure time or active commuting) [7-9].

Previous research has also established higher risk of CVD, particularly in South Asian or African descent ethnic minority groups compared to the European descent Dutch population [10]. These ethnic minority groups from South Asian or African descent constitute about one-fifth of the non-Western population in the Netherlands [11], and have lower rates of recommended physical activity with less contribution of active commuting compared to the Dutch majority population [12]. This makes them an important target group for prevention. In European-origin populations, preventive interventions often target groups in *lower* socioeconomic positions [13], as these groups are at relatively high risk of cardiovascular disease and often report lower levels of LTPA compared to the higher socioeconomic position groups [14-16]. Whether the focus of preventive interventions on persons in lower socioeconomic positions is also relevant among the South Asian and African descent ethnic minority groups is less clear.

Few studies have been carried out on this topic, and they show conflicting results: some have reported that the patterns within ethnic groups were similar to those in groups of European origin, while others found a lack of differences in socioeconomic position and LTPA [17-21]. In addition, the studies were mainly carried out among African-Americans in the United States. The situation in the European ethnic population groups may be different, as differences in national context may affect health behavior [22]. In the Netherlands, we previously found that low socioeconomic position was associated with increased risk of metabolic syndrome among European-Dutch people, but not among the South Asian and African origin ethnic minority groups [23]. Currently, it is uncertain whether such a difference in association also exists between socioeconomic position and physical activity in these ethnic groups.

Given the potential public health gain that can be achieved among these high risk populations, the aim of this study, was to assess the relationship of active commuting and LTPA with measures of socioeconomic position across two large ethnic minority groups of South Asian and African origin living in the Netherlands as compared to the European-Dutch population.

## Methods

### Study population

We used data from the population-based SUNSET (Surinamese in the Netherlands: Study on Health and Ethnicity) study, which was set up to gain insight into the cardiovascular risk profile in people 35 to 60 years of age in three ethnic groups (South Asian-Surinamese, African-Surinamese, and European-Dutch). The recruitment and design of the study have been described elsewhere [10,24]. In brief, potential participants were randomly sampled (n = 2,975) from the population register of Amsterdam, the Netherlands. Between 2001 and 2003, potential participants were approached at home for a structured face-to-face interview with a trained interviewer. The interview contained questions on lifestyle, migration history, socioeconomic position, and general health status. Ethnicity was confirmed by self-report during the interview. After the interview, participants of South Asian-Surinamese, African-Surinamese, and European-Dutch ethnicity were invited for a physical examination at a local health center. The response to the interview was 60 % in the Surinamese groups and 61 % in the European-Dutch group. All participants from the SUNSET study who provided information on ethnicity and physical activity were included in the present analysis: n = 1,626 eligible participants from the European-Dutch (n = 567), South Asian-Surinamese (n = 370), and African-Surinamese (n = 689) groups. The study was approved by the Institutional Review Board of the Academic Medical Center of the University of Amsterdam. All participants provided written informed consent.

### Physical activity

For the current analysis we focused on easily modifiable domains of activity (i.e., active commuting and LTPA). These were assessed with an adapted Short Questionnaire to Assess Health-Enhancing Physical Activity (SQUASH). The original questionnaire has been validated in European-Dutch populations, but not in ethnic minority groups [25,26]. We adapted the questionnaire, after consulting with researchers familiar with the target group, by adding some extra activities and an open-ended question identifying additional physical activity in leisure time [12]. Subsequently, the tailored questionnaire was judged on face validity by minority participants of the same background, but not further formally validated. In brief, we identified frequency, duration, and intensity of different types of major activities in the domains of active commuting (cycling and walking) and LTPA (cycling, walking, gardening, do-it-yourself activities, dancing, yoga, any other physical activity, and up to four sports). Metabolic equivalents of task (MET) values were determined based on the compendium of Ainsworth [27]. MET hours per week were calculated for active commuting and LTPA separately. MET hours per week were skewed and therefore square-root-transformed. Only moderate to vigorous activities (MET >3, non-age-specific) were included in this study, because light-intensity physical activity may be more susceptible to measurement error and response bias in subjective physical activity assessment [28]. Moreover, the focus on moderate to vigorous intensity activities will potentially reflect better the association of physical activity with health benefits.

### Socioeconomic position

Socioeconomic position was assessed in the questionnaire by self-reported highest level of education attained and occupational class. Due to the relatively low number of persons in some of the original categories, we categorized level of education as low (secondary school and below) and high (college, polytechnic, or university). Occupational class was defined as high (professionals, managers, employers, and higher grade routine non-manual employees) and low (lower grade routine non-manual, other lower grade, and skilled and unskilled manual employees) [29].

### Analysis

To assess differences in characteristics between ethnic groups, we used one-way analysis of variance for continuous variables (Kruskal-Wallis in the case of unequal variance between groups) and the chi-square test for categorical variables. Post-hoc tests with Bonferroni adjustment for multiple testing (three comparisons) were used to assess differences in characteristics.

We plotted absolute levels of physical activity in each ethnic group by socioeconomic position; this was done separately for men and for women. These figures were age-sex standardized in the ethnic groups to the age-sex distribution in the total study population (direct standardization). Linear regression models were used to assess the association between MET-hours per week, education, and occupational class. These analyses were adjusted for age and stratified by sex and ethnicity. Moreover, to calculate formal ethnicity interaction in the relationship with physical activity and socioeconomic position, we ran additional sex-stratified models, including interaction terms for ethnicity and socioeconomic position. An overall *p*-value for interaction was calculated based on the likelihood ratio test, comparing the model with and without interaction terms for socioeconomic position by ethnicity. Values of *p <* 0.05 were considered significant. Additionally, an ordinal regression analysis in categories of physical activity was considered to verify our findings from the linear regression analyses. Finally, because missing data for occupation occurred more often in the ethnic minority groups and these cases were more likely to report lower levels of active commuting and lower levels of education, we performed additional sensitivity analyses in which two extremes were considered: a situation in which all missing data for occupation was of low occupational class, and a situation in which all missing data for occupation was considered high occupational class. All analyses were performed in R version 2.15.0 (A Language and Environment for Statistical Computing, Vienna, Austria).

## Results

The European-Dutch population was older than the South Asian-Surinamese and African-Surinamese populations (Table 1). This was the case for both men and women. Level of education and occupational class were higher in European-Dutch men compared to South Asian-Surinamese and African-Surinamese men. In women, level of education and occupational class was also higher in European-Dutch women compared to South Asian-Surinamese women. Occupational class was higher in African-Surinamese women compared to South Asian-Surinamese women, but not different from European-Dutch women.

**Table 1:** Characteristics of the European-Dutch, South Asian-Surinamese, and African-Surinamese men and women included in this study

	**Men**			**Women**		
	**European-Dutch**	**South Asian-Surinamese**	**African-Surinamese**	**European-Dutch**	**South Asian-Surinamese**	**African-Surinamese**
n (%)	276 (41.3)	162 (24.2)	231 (34.5)	291 (30.4)	208 (21.7)	458 (47.9)
Age, mean (SD)	47.8 (6.7)	44.3 (6.6)^*^	43.8 (6.3)^*^	47.4 (6.8)	44.8 (6.6)^*^	43.5 (5.8)^*†^
**Education**						
Low, n (%)	**80 (29.5)**	**112 (70.0)**	**136 (59.6)**	**111 (38.3)**	**139 (68.1)**	**207 (45.8)**
High, n (%)	**191 (70.5)**	48 (30.0)^*^	92 (40.4)^*^	**179 (61.7)**	65 (31.9)^*^	**245 (54.2)**^**†**^
**Occupational class**						
Low, n (%)	**111 (40.2)**	**101 (62.3)**	**118 (51.1)**	**119 (40.9)**	**117 (56.2)**	**190 (41.5)**
High, n (%)	**160 (58.0)**	45 (27.8)^*^	83 (35.9)^*^	**162 (55.7)**	63 (30.3)^*^	**211 (46.1)**^†^
**Physical activity (MET hours/week)**						
in active commuting, median (IQR)	**0.0 (0.0-10.0)**	0.0 (0.0-0.0)*	**0.0 (0.0-5.8)**^**†**^	**0.0 (0.0-7.4)**	**0.0 (0.0-5.4)**	**2.4 (0.0-8.2)**^**†**^
transformed mean (SD)	1.6 (1.9)	0.5 (1.1)^*^	1.2 (1.5)^†^	1.4 (1.8)	1.1 (1.5)	1.6 (1.7)^**†**^
in LTPA, median (IQR)	**25.5 (10.5-47.6)**	**20.5 (5.1-39.2)**	**24.5 (9.4-51.2)**	**24.5 (11.7-38.5)**	16.6 (5.2-30.6)^*^	**19.1 (6.6-42.0)**
transformed mean (SD)	5.2 (3.0)	4.4 (3.1)^*^	5.4 (3.9)^†^	4.8 (2.5)	4.0 (2.7)*	4.5 (3.0)

MET: metabolic equivalent of task. IQR: interquartile range. SD: standard deviation. LTPA: leisure-time physical activity. Bold values represent an overall group difference (chi-square or rank-sum p < 0.05). Bonferroni adjusted group differences (3 comparisons): * different from European-Dutch (p < 0.05). ^†^ different from South Asian-Surinamese (p < 0.05).

European-Dutch and African-Surinamese men engaged more in active commuting and LTPA compared to South Asian-Surinamese men. African-Surinamese women engaged more in active commuting than South Asian-Surinamese women. European-Dutch women engaged more in LTPA compared to South Asian-Surinamese women.

**Figure 1:**
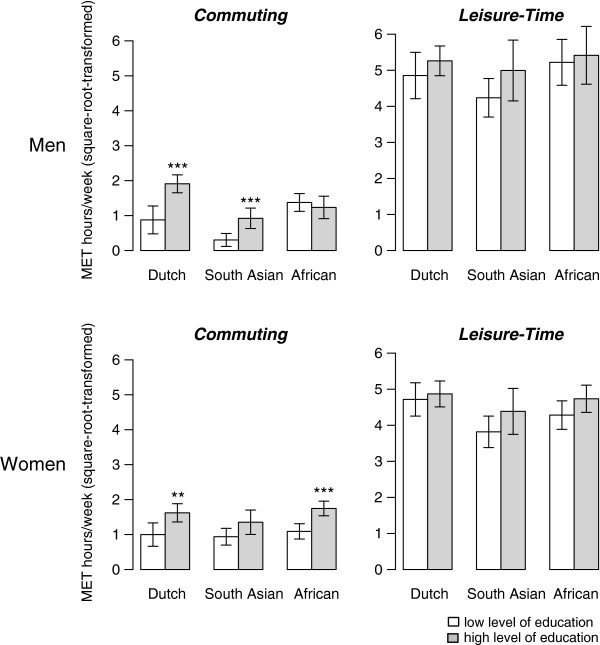
**Mean absolute levels of active commuting and leisure-time physical activity (square-root-transformed and age-sex standardized MET hours/week) in European-Dutch, South Asian-Surinamese, and African-Surinamese ethnic groups by level of education (low/high).** Statistical significance of the difference of the high compared to the low level of education group is indicated by: *** (p < 0.001), ** (p < 0.01), * (p < 0.05).

**Figure 2:**
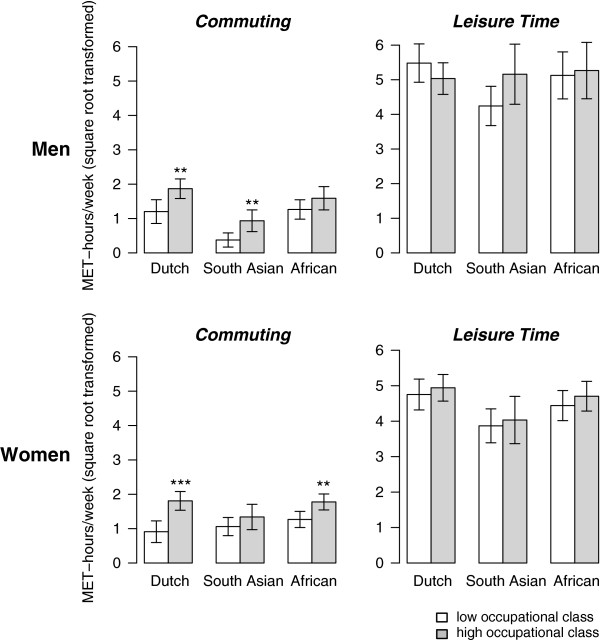
**Mean absolute levels of active commuting and leisure-time physical activity (square-root-transformed and age-sex standardized MET hours/week) in European-Dutch, South Asian-Surinamese, and African-Surinamese ethnic groups by occupational status (manual/non-manual).** Statistical significance of the difference of the non-manual and manual group is indicated by: *** (p < 0.001), ** (p < 0.01), * (p < 0.05).

Figure 1 and 2 illustrate the differences in absolute level of physical activity by measure of socioeconomic position in the ethnicity-sex groups (absolute values available in Additional file 1 and Additional file 2). For active commuting, higher level of education was positively associated with MET hours/week in European-Dutch men (beta: 0.93, 95 % CI: 0.45 1.40), and South Asian-Surinamese men (0.56, 0.19 0.92) but not in African-Surinamese men (−0.06, -0.45 0.33, *p* for interaction = 0.002, Table 2). For occupational class, the pattern of differences in active commuting across ethnic groups was similar compared to education in men, although the interaction between active commuting and ethnicity was not significant. In women, we observed the reverse for the socioeconomic position measures: there was no ethnicity interaction for active commuting by level of education, whereas we found a statistically significant differential association by ethnicity for occupational class. The gradient for active commuting and occupational class was less profound in ethnic minority women than in European-Dutch women. In European-Dutch women, active commuting was associated positively with occupational class (beta: 0.86, 95%CI: 0.44 1.28), but this was not observed in South Asian-Surinamese women (0.20, -0.25 0.66) or in African-Surinamese women (0.18, -0.15 0.50, *p* for interaction = 0.024, Table 2). The additional sensitivity analyses showed that this interaction for occupational class remained fairly stable in the extreme situation when all missing data were considered of low occupational class (*p* for interaction = 0.07) or high occupational class (*p* for interaction = 0.02, data available on request).

**Table 2:** **Association of *active commuting *in MET hours/week with education and occupational class *in men and women ***

	**Men**			***p *for interaction**	**Women**			***p *for interaction**
	**European-Dutch**	**South Asian-Surinamese**	**African-Surinamese**		**European-Dutch**	**South Asian-Surinamese**	**African-Surinamese**	
**Education**								
Low	ref = 0	ref = 0	ref = 0		ref = 0	ref = 0	ref = 0	
High, β (95 % CI)	0.93 (0.45 1.40)	0.56 (0.19 0.92)	−0.06 (−0.45 0.33)	0.002	0.53 (0.09 0.96)	0.22 (−0.22 0.65)	0.43 (0.12 0.73)	0.593
**Occupational class**								
Low	ref = 0	ref = 0	ref = 0		ref = 0	ref = 0	ref = 0	
High, β (95 % CI)	0.62 (0.17 1.07)	0.52 (0.12 0.91)	0.28 (−0.14 0.70)	0.45	0.86 (0.44 1.28)	0.20 (−0.25 0.66)	0.18 (−0.15 0.50)	0.024

MET: metabolic equivalent of task. Presented betas are based on square-root-transformed MET hours/week from moderate- and vigorous-intensity physical activity. Estimates are modeled separately for education and occupational class and adjusted for age.

The overall test for interaction is a likelihood ratio test comparing the models with and without interaction terms.

For LTPA, the association with education did not appear to significantly differ across ethnic groups among men (Table 3). For women, we also found no differences in the association between LTPA and the measures of socioeconomic position across the ethnic groups.

**Table 3:** **Association of *LTPA in MET hours/week *with education and occupational class *in men and women ***

	**Men**			***p *for interaction**	**Women**			***p *for interaction**
	**European-Dutch**	**South Asian-Surinamese**	**African-Surinamese**		**European-Dutch**	**South Asian-Surinamese**	**African-Surinamese**	
**Education**								
Low	ref = 0	ref = 0	ref = 0		ref = 0	ref = 0	ref = 0	
High, β (95 % CI)	0.41 (−0.35 1.17)	1.03 (−0.00 2.06)	−0.01 (−1.04 1.02)	0.377	0.13 (−0.48 0.73)	0.72 (−0.08 1.51)	0.20 (−0.35 0.75)	0.536
**Occupational class**								
Low	ref = 0	ref = 0	ref = 0		ref = 0	ref = 0	ref = 0	
High, β (95 % CI)	−0.36 (−1.09 0.36)	0.92 (−0.17 2.00)	−0.27 (−1.33 0.79)	0.16	0.22 (−0.36 0.81)	0.13 (−0.72 0.97)	0.06 (−0.53 0.64)	0.93

LTPA: leisure-time physical activity. MET: metabolic equivalent of task. Presented betas are based on square-root-transformed MET hours/week from moderate- and vigorous-intensity physical activity. Estimates are modeled separately for education and occupational class and adjusted for age.

The overall test for interaction is a likelihood ratio test comparing the models with and without interaction terms.

The results from the ordinal regression analysis were consistent with the associations and interactions presented above (Additional file 3).

## Discussion

### Main findings

We found a positive association between socioeconomic position and physical activity, in terms of active commuting and LTPA. However, the association between socioeconomic position and active commuting differed across ethnic groups. In men, we found a positive association with socioeconomic position (in particular for high level of education) in the European-Dutch, which was less strong in South Asian-Surinamese and African-Surinamese men. Among women we observed a similar pattern of interaction for socioeconomic position (but then for occupational class), with a positive association in the European-Dutch women and less strong associations in the South Asian-Surinamese and African-Surinamese women.

For LTPA, among both men and women, we were unable to observe a clear pattern of differences in the association with socioeconomic position between the ethnic groups.

Our study shows that active commuting appears to be less strongly related to socioeconomic position across ethnic minority groups compared to the positive association in the European-Dutch. This was most apparent among the African-Surinamese. We are unaware of any earlier population studies that focused specifically on the relationship of active commuting and measures of socioeconomic position across multiple ethnic groups.

The weaker socioeconomic gradient with active commuting in the Surinamese compared to the European-Dutch could be related to the relatively higher number of European-Dutch with high levels of education [30], which was not captured by our measure of socioeconomic position.

Another possible explanation for the observed differential association between active commuting and socioeconomic position across ethnic minority groups is a difference in preferred mode of transportation. We speculate that the higher level of active commuting in the high socioeconomic position European-Dutch compared to their low socioeconomic position counterparts may be related to the frequency of bicycle use in this population compared to other groups [12,31], while the observation of a lack of association between low and high socioeconomic position for active commuting with African-Surinamese groups might be related to a preference for walking or using public transportation for their commutes [12,31,32]. Environmental factors, such as neighborhood factors, car/bike ownership and distance to employment, could be an important explanation for the observed patterns between the ethnic groups. However, these factors were not assessed in this study. More research is clearly necessary which takes into account the influence of such environmental factors.

For LTPA, we observed no clear differential association with socioeconomic position across ethnic minority groups. In our study, the low socioeconomic groups from all backgrounds engaged in levels of LTPA that were comparable to their high socioeconomic position counterparts; this diminishes the likelihood of a socioeconomic gradient. A lack of association between LTPA and socioeconomic position has been reported before [33]. However, other studies have found an ethnicity interaction in the relationship between LTPA and socioeconomic position [34] or associations of LTPA and socioeconomic position, also in ethnic minority groups [18,19]. The difference between these studies and our own might be related to the socioeconomic context in which the studies were carried out. In our study, the low socioeconomic position groups engaged in relatively high levels of LTPA (compared to their high socioeconomic position counterparts), which diminishes the likelihood of a socioeconomic gradient. One methodological difference between our study and that of others potentially relating to this finding was the broad measurement of physical activity in our study. The focus of our questionnaire was to measure habitual activity in leisure time, in addition to measure participation in sports and exercise in leisure time. In other studies, and of course depending on the reasons for the inquiry, sports and exercise were often the only group of activities identified from the leisure time domain [35]. The habitual activity (e.g., walking, cycling, gardening, dancing, exercising at home) additionally measured in our study in LTPA could be less constrained by barriers (e.g., financial, or facilities within range) often reported in low socioeconomic position groups. Therefore, this could have resulted in higher levels of measured physical activity during leisure time in the low socioeconomic position group in our study; which potentially diminishes the socioeconomic gradient in LTPA. Additionally, results from qualitative studies indicate that those in low socioeconomic position groups and ethnic minority groups are aware of the beneficial health aspects of LTPA and exercise, which could also have mitigated the socioeconomic gradient in our study population [36-38].

Finally, although not as strong as the European-Dutch gradient, the positive gradient in active commuting for the South Asian-Surinamese and the lack of ethnicity interaction in the relationship between socioeconomic position and LTPA fits with the convergence hypothesis that the socioeconomic gradient in the South Asian-Surinamese and African-Surinamese ethnic minority groups may change in the direction of European-Dutch levels. This is in line with the observation by Bos et al. that the overall mortality pattern in Surinamese men and women and cardiovascular mortality in Surinamese women compared to the European-Dutch may indicate convergence of health-behavior with increasing duration of residence [39]. Additionally, Nierkens et al. suggested that the Dutch Surinamese population, especially men, have already reversed the socioeconomic gradient in smoking behavior that would normally be expected according to the stages of the tobacco epidemic [40]. In the Indian ethnic minority group living in the United Kingdom (UK), convergence towards the majority population of a wide range of health behaviors is also observed [41,42], this group can be characterized with an even longer average duration of residence compared to other South Asian ethnic minority groups in the UK, and the South Asian- and African-Surinamese in the Netherlands [43].

Given these examples, it seems probable that in the long term the physical activity patterns (also for active commuting) observed in the South Asian-Surinamese and African-Surinamese populations will converge towards European averages. Especially in the case of socioeconomic advancement in the Surinamese groups, this balance is expected to tip in favor of a more European pattern of socioeconomic gradient. To be able to observe such trends, though, longitudinal data on physical activity and socioeconomic position within the ethnic groups is a prerequisite.

### Limitations

Socioeconomic position is a complex social construct for which various indicators have been shown to capture different aspects of importance in relation to the outcome under study [44]. In the Netherlands, education is identified as a stable indicator of socioeconomic position [45]. Because of the small numbers per group in some of the original categories, we were limited to a relatively crude high-low comparison. As a result we might have missed subgroup patterns. Therefore, we presented an additional measure of socioeconomic position, namely based on occupational class. We did not include other measures of socioeconomic position (such as income), which could have provided additional insights into the association of socioeconomic position and physical activity across ethnic minority groups. For example, higher income could enable easier access to facilities [44].

We were unable to take into account processes of acculturation in this study, while acculturation processes in the migrant population are also expected to contribute to the convergence of physical activity behavior towards the majority population in a country [46]. Duration of residence was measured and previously used as proxy of acculturation [22], but not included in this analyses because of a lack of variation observed in our study population. The majority of the ethnic minority individuals in our study population have a relative long duration of residence, which makes such an analysis less informative.

Furthermore, self-reported levels of physical activity are prone to reporting bias or cognitive bias [47], resulting in measurement error that could have influenced the presented findings. Another point relating to our measurement of physical activity is that we focused on moderate to vigorous physical activity, which is expected to capture the relationship with health, but excluded light walking activity, which may also have a beneficial effect on health. Moreover, the focus on LTPA and active commuting meant that we could not take into account potential socio-economic differences in occupational activity [48], however in our study population the relationship between leisure-time/active commuting and occupational physical activity was similar across ethnic groups (unpublished). Additionally, there might be cross-cultural differences in reporting levels of physical activity [49]. Since we were mainly interested in the association of physical activity *within* each ethnic minority group, we believe that cross-cultural differences in reporting might be less of a problem in this study.

Finally, we acknowledge that the generalizability of the findings presented in this study may be limited to the included ethnic groups and context. The data were collected between 2001 and 2003. Since then, circumstances may have changed. However, the data presented are relevant, as more recent studies, and studies including more information on environmental factors, are unfortunately not yet available for these ethnic minority groups in the Netherlands. Additionally, this study reported on cross-sectional study data, which means that any causal inference from this data should be made with caution. Other patterns in active commuting or LTPA by socioeconomic position might exist in those from ethnic minority groups of similar descent who live elsewhere or with a different migration history.

## Conclusion

Our study suggests that the positive socioeconomic gradient in active commuting observed in the European-Dutch may be less strong in the South Asian- and African-Surinamese ethnic groups. This was not observed for LTPA. The independent effect of ethnicity on active commuting was observed for men and women, but this was dependent on the measure of socioeconomic position used.

Our findings imply that, contrary to the typical targeting of physical activity interventions to the lower socioeconomic groups often applied in European-origin populations, a broader focus may be needed for activities or recommendations for physical activity among African- and South Asian-origin groups. Specifically, public health workers should be aware of the current low levels of active commuting in both low and high socioeconomic position groups from these populations. Based on this, it seems relevant to stimulate active commuting among the African-Surinamese and South Asian-Surinamese in the Netherlands in both low and high socioeconomic position groups.

## Abbreviations

LTPA: Leisure-time physical activity; MET: Metabolic equivalents of task; SUNSET: Surinamese in the Netherlands: Study on Health and Ethnicity; SQUASH: Short Questionnaire to Assess Health-Enhancing Physical Activity.

## Competing interests

The authors declare that theyhave no competing interests.

## Authors’ contributions

JM designed the study, carried out the data analysis and interpretation, and drafted the first version of the manuscript. LB participated in interpretation of data and critical revisions of the manuscript. CA, KS, IV contributed to the conception and design of the study, participated in interpretation of data and critical revisions of the manuscript. All authors approved the final version of this manuscript.

## Pre-publication history

The pre-publication history for this paper can be accessed here:

http://www.biomedcentral.com/1471-2458/12/815/prepub

## Supplementary Material

Additional file 1 Active commuting and LTPA in MET hours/week by level of education in men and women. Click here for file

Additional file 2 Active commuting and LTPA in MET hours/week by occupation in men and women. Click here for file

Additional file 3 **Association of *active commuting *with education and occupational class *in men and women. *** Click here for file
