# Supplementary material for: The association of leisure-time physical activity and active commuting with measures of socioeconomic position in a multiethnic population living in the Netherlands: results from the cross-sectional SUNSET study
Source: BMC Public Health. 2012 Sep 21;12:815. doi: 10.1186/1471-2458-12-815 (PMC3490879; doi:10.1186/1471-2458-12-815)
Supplement: Additional file 1 — Active commuting and LTPA in MET hours/week by level of education in men and women. [file 1471-2458-12-815-S1.doc]

| Additional file 1. Active commuting and LTPA in MET hours/week by **level of education** in men and women | | | | | | |
| --- | --- | --- | --- | --- | --- | --- |
| **Men** | **European-Dutch** |  | **South Asian-Surinamese** |  | **African-Surinamese** |  |
|  | Low | High | Low | High | Low | High |
| Active commuting | 0.88 (0.48-1.28) | **1.91 (1.65-2.17)** | 0.30 (0.12-0.49) | **0.92 (0.63-1.21)** | 1.38 (1.12-1.63) | 1.23 (0.91-1.56) |
| Leisure time | 4.85 (4.21-5.50) | 5.26 (4.85-5.67) | 4.24 (3.70-4.77) | 4.99 (4.15-5.84) | 5.22 (4.58-5.86) | 5.41 (4.61-6.22) |
|  |  |  |  |  |  |  |
| **Women** |  |  |  |  |  |  |
|  |  |  |  |  |  |  |
| Active commuting | 1.00 (0.66-1.33) | **1.62 (1.36-1.88)** | 0.94 (0.70-1.18) | 1.35 (1.00-1.70) | 1.09 (0.87-1.31) | **1.75 (1.54-1.96)** |
| Leisure time | 4.72 (4.25-5.18) | 4.87 (4.51-5.23) | 3.82 (3.38-4.26) | 4.39 (3.75-5.02) | 4.28 (3.89-4.68) | 4.73 (4.36-5.11) |
|  |  |  |  |  |  |  |
| LTPA: leisure-time physical activity. MET: metabolic equivalent of task. Presented values are mean MET hours/week and are square-root-transformed and age-sex standardized (direct standardization) to total study population. Values in bold indicate statistical significant difference compared to low group (*p*<0.05). | | | | | | |
