# Supplementary material for: The association of leisure-time physical activity and active commuting with measures of socioeconomic position in a multiethnic population living in the Netherlands: results from the cross-sectional SUNSET study
Source: BMC Public Health. 2012 Sep 21;12:815. doi: 10.1186/1471-2458-12-815 (PMC3490879; doi:10.1186/1471-2458-12-815)
Supplement: Additional file 2 — Active commuting and LTPA in MET hours/week by occupation in men and women. [file 1471-2458-12-815-S2.doc]

| Additional file 2. Active commuting and LTPA in MET hours/week by **occupation** in **men and women** | | | | | | |
| --- | --- | --- | --- | --- | --- | --- |
| **Men** | **European-Dutch** |  | **South Asian-Surinamese** |  | **African-Surinamese** |  |
|  | Manual | Non-Manual | Manual | Non-Manual | Manual | Non-Manual |
| Active Commuting | 1.03 (0.66-1.41) | **1.89 (1.62-2.16)** | 0.35 (0.14-0.55) | **0.95 (0.65-1.24)** | 1.35 (1.04-1.66) | 1.44 (1.14-1.75) |
| Leisure time | 5.81 (5.21-6.41) | **4.91 (4.48-5.34)** | 4.23 (3.65-4.81) | 5.10 (4.27-5.93) | 5.15 (4.40-5.89) | 5.22 (4.49-5.95) |
|  |  |  |  |  |  |  |
| **Women** |  |  |  |  |  |  |
|  |  |  |  |  |  |  |
| Active Commuting | 0.70 (0.35-1.05) | **1.80 (1.55-2.05)** | 0.95 (0.62-1.28) | 1.31 (1.03-1.59) | 1.20 (0.86-1.53) | **1.63 (1.44-1.83)** |
| Leisure time | 4.79 (4.31-5.28) | 4.89 (4.54-5.24) | 3.75 (3.16-4.35) | 4.05 (3.54-4.56) | 4.32 (3.73-4.92) | 4.66 (4.31-5.00) |
|  |  |  |  |  |  |  |
| LTPA: leisure-time physical activity. MET: metabolic equivalent of task. Presented values are mean MET hours/week and are square-root-transformed and age-sex standardized (direct standardization) to total study population. Values in bold indicate statistical significant difference compared to low group (*p*<0.05). | | | | | | |
