# Supplementary material for: The association of leisure-time physical activity and active commuting with measures of socioeconomic position in a multiethnic population living in the Netherlands: results from the cross-sectional SUNSET study
Source: BMC Public Health. 2012 Sep 21;12:815. doi: 10.1186/1471-2458-12-815 (PMC3490879; doi:10.1186/1471-2458-12-815)
Supplement: Additional file 3 — Association of active commuting with education and occupational class in men and women. [file 1471-2458-12-815-S3.doc]

Data supplement 3

Association of ***active commuting*** with education and occupational class ***in men and women***.

| Education |  |  | β | S.E. | *p*-value |
| --- | --- | --- | --- | --- | --- |
| Men | Dutch | Low | reference=0 |  |  |
|  |  | High | 0.9754 | 0.2712 | 0.0003 |
|  | South Asian | Low | reference=0 |  |  |
|  |  | High | 1.0394 | 0.3992 | 0.0092 |
|  | African | Low | reference=0 |  |  |
|  |  | High | -0.0370 | 0.2629 | 0.8882 |
|  |  |  |  |  |  |
| Women | Dutch | Low | reference=0 |  |  |
|  |  | High | 0.5754 | 0.2399 | 0.0165 |
|  | South Asian | Low | reference=0 |  |  |
|  |  | High | 0.2390 | 0.2968 | 0.4207 |
|  | African | Low | reference=0 |  |  |
|  |  | High | -0.1026 | 0.1786 | 0.5656 |
|  |  |  |  |  |  |
| Occupational class |  |  | β | S.E. | *p*-value |
| Men | Dutch | Low | reference=0 |  |  |
|  |  | High | 0.5637 | 0.2388 | 0.0182 |
|  | South Asian | Low | reference=0 |  |  |
|  |  | High | 0.7602 | 0.4011 | 0.0580 |
|  | African | Low | reference=0 |  |  |
|  |  | High | 0.4527 | 0.2786 | 0.1042 |
|  |  |  |  |  |  |
| Women | Dutch | Low | reference=0 |  |  |
|  |  | High | 0.9109 | 0.2420 | 0.0002 |
|  | South Asian | Low | reference=0 |  |  |
|  |  | High | 0.1826 | 0.3041 | 0.5482 |
|  | African | Low | reference=0 |  |  |
|  |  | High | 0.2459 | 0.1877 | 0.1901 |

Values are coefficients from ordinal regression analyses. Outcome was defined as moderate and vigorous intensity physical activity and was measured in MET-hours/week in 3 categories (0, 0-10, and 10+ MET).

Association of ***leisure time physical activity*** with education and occupational class ***in men and women***.

| Education |  |  | β | S.E. | *p*-value |
| --- | --- | --- | --- | --- | --- |
| Men | Dutch | Low | reference=0 |  |  |
|  |  | High | 0.3442 | 0.2985 | 0.2490 |
|  | South Asian | Low | reference=0 |  |  |
|  |  | High | 0.7384 | 0.3793 | 0.0515 |
|  | African | Low | reference=0 |  |  |
|  |  | High | 0.2033 | 0.3036 | 0.5030 |
|  |  |  |  |  |  |
| Women | Dutch | Low | reference=0 |  |  |
|  |  | High | 0.4517 | 0.2785 | 0.1048 |
|  | South Asian | Low | reference=0 |  |  |
|  |  | High | 0.3361 | 0.3243 | 0.2999 |
|  | African | Low | reference=0 |  |  |
|  |  | High | 0.4155 | 0.1971 | 0.0351 |
|  |  |  |  |  |  |
| Occupational class |  |  | β | S.E. | *p*-value |
| Men | Dutch | Low | reference=0 |  |  |
|  |  | High | 0.3846 | 0.2844 | 0.1762 |
|  | South Asian | Low | reference=0 |  |  |
|  |  | High | 0.4918 | 0.3892 | 0.2063 |
|  | African | Low | reference=0 |  |  |
|  |  | High | 0.3735 | 0.3255 | 0.2511 |
|  |  |  |  |  |  |
| Women | Dutch | Low | reference=0 |  |  |
|  |  | High | 0.3595 | 0.2837 | 0.2050 |
|  | South Asian | Low | reference=0 |  |  |
|  |  | High | -0.1905 | 0.3209 | 0.5528 |
|  | African | Low | reference=0 |  |  |
|  |  | High | 0.0076 | 0.2082 | 0.9710 |

Values are coefficients from ordinal regression analyses. Outcome was defined as moderate and vigorous intensity physical activity and was measured in MET-hours/week in 3 categories (0, 0-10, and 10+ MET).
